# Positive Effects of Allicin on Cytotoxicity, Antioxidative Status, and Immunity in “*Eriocheir sinensis*” Hepatopancreatic Cells Against Oxidative Stress-Induced Injury

**DOI:** 10.3390/antiox15010093

**Published:** 2026-01-12

**Authors:** Yiqing Guo, Peng Huang, Wenhui Wang, Jingwen Wu, Jinliang Du, Jiayi Li, Jiancao Gao, Haojun Zhu, Jun Gao, Yao Zheng, Yanbing Zhuang, Gangchun Xu, Liping Cao

**Affiliations:** 1 Wuxi Fisheries College, Nanjing Agricultural University, Wuxi 214081, China; 2023813026@stu.njau.edu.cn (Y.G.); 2024213005@stu.njau.edu.cn (P.H.); 2024813025@stu.njau.edu.cn (J.W.); dujl@ffrc.cn (J.D.); gaojiancao@ffrc.cn (J.G.); zhuhaojun@ffrc.cn (H.Z.); gaojun@ffrc.cn (J.G.); zhengyao@ffrc.cn (Y.Z.); 2 College of Fisheries and Life Science, Dalian Ocean University, Dalian 116023, China; holly0907@163.com; 3 Freshwater Fisheries Research Center, Chinese Academy of Fishery Sciences, Wuxi 214081, China; lijiayi@ffrc.cn (J.L.); zyb19910820@163.com (Y.Z.)

**Keywords:** Nrf2, p38-MAPK, NF-κB, T-BHP, cell culture

## Abstract

Oxidative stress represents a critical threat to aquatic animal health and aquaculture productivity. Allicin, a natural plant extract, has not been systematically investigated for its antioxidant mechanisms in aquatic crustaceans. This study established in vitro and in vivo models of tert-butyl hydroperoxide (T-BHP)-induced oxidative stress in Chinese mitten crabs (*Eriocheir sinensis*) to evaluate the hepatoprotective effects of allicin. Integrating biochemical, transcriptomic, and ultrastructural analyses, we found that allicin significantly alleviated T-BHP-induced cytotoxicity and oxidative damage in vitro. Mechanistically, allicin up-regulated antioxidant genes including glutathione peroxidase (*gpx*) and thioredoxin reductase 1 (*trxr1*), and down-regulated pro-inflammatory cytokines such as interleukin-1 beta (*il-1β*), suggesting the concomitant activation of the Nrf2 signaling pathway and inhibition of the p38-MAPK/NF-κB pathway. Transcriptomics further indicated its role in restoring proteostasis and mitochondrial function. A 35-day feeding trial validated these findings in vivo; dietary supplementation with 300 mg·kg^−1^ allicin effectively reversed T-BHP-induced disturbances in antioxidant enzyme activities and immune-related gene expression. These consistent findings demonstrate that allicin alleviates hepatopancreatic oxidative damage through multi-pathway synergism, supporting its potential as a green and effective antioxidant feed additive in aquaculture.

## 1. Introduction

Oxidative stress represents a pathophysiological process characterized by an imbalance between the production and elimination of reactive oxygen species (ROS) under harmful stimuli, leading to disruption of the prooxidant-antioxidant homeostasis and subsequent damage to cellular structures and functions [1]. The Chinese mitten crab constitutes an economically significant aquaculture species in China, with continuously expanding production scale reaching 894,395 tons in 2024 [2]. In intensive aquaculture systems, multiple environmental stressors including temperature fluctuations, dissolved oxygen deficiency, ammonia accumulation, salinity-alkalinity variations, pathogenic microorganisms, heavy metal contamination, pesticide residues, and anthropogenic activities can induce oxidative stress [3,4,5,6,7], resulting in diminished growth performance, immunosuppression, and elevated mortality rates, thereby causing substantial economic losses. Consequently, developing efficient and environmentally friendly antioxidant strategies has emerged as a crucial research direction in aquaculture.

Although synthetic antioxidants such as butylated hydroxyanisole (BHT), butylated hydroxytoluene (BHA), and tertiary butylhydroquinone (TBHQ) are widely employed, excessive application or improper use may induce carcinogenicity, cytotoxicity, oxidative stress induction, and endocrine disruption [8,9]. Their potential food safety concerns and environmental residues warrant serious attention. In this context, exploring safe and effective antioxidant alternatives from natural plant sources carries significant theoretical and practical importance. Research indicates that curcumin extracted from turmeric (*Curcuma longa*) exhibits remarkable antioxidant, anti-inflammatory, and hepatoprotective properties. Dietary supplementation with appropriate doses of curcumin enhances antioxidant capacity and immune function in rainbow trout (*Oncorhynchus mykiss*), Nile tilapia (*Oreochromis niloticus*), and crucian carp (*Carassius auratus*) [10]. Rutin isolated from Chinese toon (*Toona sinensis*) serves as an immunostimulant in white-leg shrimp (*Litopenaeus vannamei*), with injected rutin significantly improving antioxidant status and strengthening immune defense against *Vibrio alginolyticus* infection [11]. Furthermore, icariin (ICA) derived from Epimedium species demonstrates outstanding antioxidant, immunoprotective, and anti-inflammatory activities. Dietary supplementation with 100 mg·kg^−1^ ICA significantly enhances antioxidant capacity in Chinese mitten crabs and alleviates lipopolysaccharide (LPS)-induced hepatopancreatic oxidative damage [12].

Allicin, an organosulfur compound extracted from garlic (*Allium sativum*), has attracted considerable attention due to its broad-spectrum antimicrobial, anti-inflammatory, and antioxidant activities [13,14,15]. Previous studies have confirmed that allicin effectively enhances immune function, improves antioxidant capacity, and modulates inflammatory responses and apoptosis processes [16,17,18]. Currently, allicin finds relatively widespread application in livestock production. Research reports indicate that allicin alleviates LPS-induced inflammatory responses in bovine mammary epithelial cells by suppressing the TLR4/NF-κB signaling pathway. In LPS-pretreated cell models, inflammatory cytokines including interleukin-1 beta (IL-1β), IL-6, and tumor necrosis factor-alpha (TNF-α) were significantly elevated, while 1 μM and 2.5 μM allicin treatments effectively inhibited their expression, with 2.5 μM allicin demonstrating optimal efficacy [18]. Another study confirmed that dietary allicin supplementation ameliorated lead-induced abnormalities in hepatic antioxidant parameters (including reduced glutathione (GSH), catalase (CAT), superoxide dismutase (SOD), and total antioxidant capacity (T-AOC)) in chickens and alleviated lead poisoning symptoms via the PI3K signaling pathway [19]. Additionally, dietary supplementation with 1 g·kg^−1^ allicin in tilapia reversed furan-induced alterations in CAT, SOD, T-AOC, and malondialdehyde (MDA) levels, significantly reducing furan toxicity and oxidative damage [20]. However, research on allicin in aquatic animals, particularly crustaceans such as Chinese mitten crabs, remains relatively limited, with current reports confined to species including kuruma shrimp (*Marsupenaeus japonicus*) [21], white-leg shrimp [22], and black tiger shrimp (*Penaeus monodon*) [23].

T-BHP, a classic organic peroxide, is widely utilized to establish both in vitro and in vivo oxidative stress models due to its propensity to decompose and generate free radicals, thereby initiating robust oxidative reactions [24,25]. In biomedical research, T-BHP-induced models serve as a pivotal tool for investigating oxidative damage mechanisms and evaluating the effects of potential protective agents. For instance, in cardiomyocyte lines such as AC16 and H9c2, T-BHP exposure induces ROS generation, with the resulting cell mortality showing a concentration-dependent relationship that varies between different cell lines [26]. Furthermore, studies have demonstrated that pharmacological preconditioning (e.g., with morphine) can significantly enhance H9c2 cell viability by mitigating T-BHP-induced ROS accumulation, protein carbonylation, and lipid peroxidation [27]. These findings collectively validate the efficacy of the T-BHP model for dissecting antioxidant defense systems. Consequently, this model is extensively employed in screening and assessing the antioxidant capacity of various compounds, such as evaluating the protective effects of acteoside and its derivatives on HepG2 cells [28]. However, existing research employing the T-BHP model has predominantly focused on mammalian cellular systems. Its application and the investigation of corresponding protective mechanisms remain insufficient in aquatic invertebrates, particularly in economically crucial crustaceans.

This study established an in vitro oxidative stress model in hepatopancreatic cells of Chinese mitten crabs induced by T-BHP. Integrating biochemical assays, ultrastructural examination, and transcriptomic profiling, we systematically investigated T-BHP-induced hepatopancreatic oxidative damage and the antioxidant protective mechanisms of allicin at the cellular level, aims to test the potential antioxidant defense and immunomodulatory mechanisms of allicin, such as those involving the Nrf2 and NF-κB signaling pathways. Concurrently, we conducted a 35-day feeding trial to validate the preventive effects of allicin against oxidative stress in vivo through dietary supplementation with varying allicin concentrations, and determined the optimal dosage, thereby providing scientific evidence for the practical application of allicin in preventing and controlling oxidative damage in aquaculture.

## 2. Materials and Methods

### 2.1. Experimental Animals and Husbandry

Juvenile Chinese mitten crabs with an initial body weight of 12 ± 0.5 g were obtained from the Yangzhong Base of the Freshwater Fisheries Research Center, Chinese Academy of Fishery Sciences. During the experimental period, crabs were maintained in aquaria (970 mm × 460 mm × 240 mm) with water temperature stabilized at 23 °C, dissolved oxygen concentration exceeding 7 mg·L^−1^, pH 6.5–8.5, ammonia nitrogen < 0.2 mg·L^−1^, TDS < 450 mg·L^−1^, nitrite < 0.005 mg·L^−1^, and H_2_S < 0.02 mg·L^−1^. Half of the water volume was replaced daily at 9:00 AM using aerated tap water (aerated for 24 h prior to use), and formulated feed was provided at 16:00 PM at a rate of 1% of total body weight. The photoperiod was set at 14 h of light and 10 h of darkness (L:D = 14:10). This study was approved by the Animal Care and Use Committee of Nanjing Agricultural University (Nanjing, China) (SYXK 2022-0031). All animal procedures were performed according to the Guideline for the Care and Use of Laboratory Animals in China.

### 2.2. Materials and Reagents

Allicin (purity ≥ 98%) was purchased from Wuhan Tianzhi Biotechnology Co., Ltd. (Wuhan, China). Tert-butyl hydroperoxide (T-BHP) was acquired from Aladdin Reagent (Shanghai) Co., Ltd. (Shanghai, China). The CCK-8 cell viability assay kit was obtained from APExBIO (Shanghai Weihuan Biotechnology Co., Ltd., Shanghai, China. catalog number: K1018). L-15 medium and penicillin-streptomycin solution were supplied by Sigma-Aldrich (St. Louis, MO, USA). Fetal bovine serum (FBS) was sourced from GIBCO (Grand Island, NY, USA). All cell culture plates were products of Corning Incorporated (Corning, NY, USA). The MiniBEST Universal RNA Extraction Kit, PrimeScript^TM^ 1st Strand cDNA Synthesis Kit, and TB Green Fast qPCR mix were procured from TaKaRa (Baoriyi Biotechnology (Beijing) Co., Ltd., Beijing, China). acid phosphatase (ACP, catalog number: A060-2), alkaline phosphatase (AKP, A059-2), glutamic pyruvic transaminase (GPT, C009-2-1), CAT (A007-1-1), T-AOC (A015-2-1), oxidized glutathione (GSSG, A061-1-2), MDA (A003-2), glutamic oxaloacetic transaminase (GOT, C010-2-1), GSH (A006-2-1), total superoxide dismutase (T-SOD, A001-3), and total protein (TP, A045-2) were purchased from Nanjing Jiancheng Bioengineering Co., Ltd. (Nanjing, Jiangsu, China). All other chemicals used were of analytical grade. Expanded formulated feed for crabs was supplied by Jiangsu Tongwei Biotechnology Co., Ltd. (Hai’an, Jiangsu, China).

### 2.3. Isolation and Culture of Hepatopancreatic Primary Cells

After one week of acclimation, healthy and active crabs were selected for primary cell isolation. Following disinfection in 1:5000 potassium permanganate solution for 30 min and surface sterilization with 75% ethanol, crabs were transferred to a biosafety cabinet for aseptic dissection. Hepatopancreatic tissues were excised, rinsed 2–3 times with PBS, and meticulously minced before filtration to obtain cell suspensions. After centrifugation at 2000 r·min^−1^ for 10 min at 4 °C, the supernatant was discarded. Cell pellets were resuspended in L-15 complete medium (supplemented with 5 μg·mL^−1^ amphotericin B, 200 IU·mL^−1^ penicillin-streptomycin, and 5% FBS). Cell density was adjusted to 1 × 10^5^ cells·mL^−1^ using a hemocytometer, and 100 μL aliquots were seeded per well in 96-well plates. Cultures were maintained at 28 °C for 12 h prior to subsequent experiments.

### 2.4. Determination of In Vitro Concentrations of Allicin and T-BHP (CCK-8 Assay)

Following the optimization of hepatopancreatic cell density, the cells were assigned to the following treatment groups: the blank group (wells containing complete L-15 medium only, without cells or any reagents), the control group (wells seeded with cells in complete L-15 medium, without further treatment), and six experimental groups (wells seeded with cells in complete L-15 medium and then treated with allicin at final concentrations of 2, 4, 8, 16, 32, and 64 μg·mL^−1^, respectively). A stock solution of allicin was prepared at 40 mg·mL^−1^ by dissolving 40 mg of allicin in 1 mL of dimethyl sulfoxide (DMSO), which was subsequently serially diluted with L-15 medium to obtain the required working concentrations (0.005–0.16% DMSO *v*/*v*). Preliminary experiments indicated that 0.16% DMSO (corresponding to 64 μg·mL^−1^ allicin) had no effect on the viability of hepatopancreatic cells compared to the DMSO-free control group [29]. All abovementioned experiments were repeated six times. All culture plates were subsequently transferred to an incubator maintained at 28 °C for 24 h, after which cell viability was assessed to determine the safe concentration range of allicin.

For the oxidative stress modeling, T-BHP was dissolved in PBS buffer. In this experiment, the blank and control groups were treated as described above and cultured for a total of 28 h. For the six T-BHP treatment groups, cells were seeded and cultured for 24 h as described, followed by exposure to T-BHP at different final concentrations (6.25, 12.5, 25, 50, 100, and 200 μM) for 4 h. All abovementioned experiments were repeated six times. Cell viability was then measured to determine the optimal T-BHP concentration for establishing the oxidative stress model.

For the determination of the optimal concentration of allicin, the experimental setup was as follows: Control group: cultured in L-15 complete medium for 28 h; Model group: cultured in L-15 complete medium for 24 h, then replaced with medium containing a final concentration of 25 μM T-BHP for an additional 4 h; Allicin treatment groups: cultured in medium containing 0.25, 0.5, 1, 2, or 4 μg·mL^−1^ allicin for 24 h, followed by replacement with medium containing 25 μM T-BHP for oxidative stress induction over 4 h. All abovementioned experiments were repeated six times. Cell viability was subsequently assessed to determine the optimal protective concentration of allicin.

Cell viability assay: 10 μL of CCK-8 solution was added to each well, followed by further incubation for 2 h. The absorbance at a wavelength of 450 nm was then measured and recorded using a microplate reader (SpectraMax M5, Molecular Devices, San Jose, CA, USA).

Cell viability (%) = [(ODexperimental − ODblank)/(ODcontrol − ODblank)] × 100%

where: ODexperimental represents the absorbance of the experimental group; ODblank represents the absorbance of the blank group; ODcontrol represents the absorbance of the control group.

### 2.5. In Vitro Investigation of Protective Mechanisms

Four experimental groups were established: control group (CG) and model group (T-BHP), which were treated as described in Section 2.4 and cultured for 28 h; allicin control group (AC, with 2 μg·mL^−1^ allicin for 28 h), and allicin protection group (AC-T, pretreated with 2 μg·mL^−1^ allicin for 24 h followed by 25 μM T-BHP exposure for 4 h). All abovementioned experiments were repeated six times. Cell supernatants and pellets were collected for biochemical analysis, gene expression profiling, transcriptome sequencing, and ultrastructural observation.

#### 2.5.1. Cell Supernatants Biochemical Analysis

Following manufacturer’s protocols, cell supernatants were directly used for the determination of ACP (analysis wavelength: 520 nm), AKP (520 nm), GPT (505 nm), CAT (405 nm), T-AOC (405 nm), GSSG (405 nm), and MDA (532 nm) levels.

#### 2.5.2. Ultrastructural Examination

Cell pellets were washed three times with phosphate-buffered saline (PBS) and sequentially fixed with 2.5% glutaraldehyde in 0.1 M phosphate buffer (pH 7.4) for 4 h at 4 °C, followed by post-fixation with 1% osmium tetroxide in the same buffer for 2 h at 4 °C. After fixation, the samples were rinsed with PBS and then dehydrated through a graded series of ethanol (50%, 70%, 80%, 90%, 95%, and 100%) for 15 min each, followed by two changes in absolute acetone for 15 min each. The dehydrated samples were subsequently infiltrated and embedded in Epon 812 resin. Ultrathin sections (approximately 70–90 nm in thickness) were cut using a diamond knife on an ultramicrotome, collected on copper grids, and double-stained with uranyl acetate and lead citrate to enhance contrast. Cellular ultrastructure was examined using transmission electron microscopy (Talos F200C, Thermo Fisher Scientific, Waltham, MA, USA).

#### 2.5.3. Gene Expression Analysis

Total RNA was extracted using the MiniBEST kit, and cDNA was synthesized from qualified RNA samples. Amplification reactions were performed using TB Green Fast qPCR mix on a CFX96 RT-PCR system under the following conditions: 95 °C for 30 s; 40 cycles of 95 °C for 5 s and 60 °C for 30 s. Reaction specificity was verified through melting curve analysis. Relative gene expression was calculated using the 2^−ΔΔCt^ method with *s27* as the internal control. All primers were synthesized by Yixin Biotechnology (Shanghai) Co., Ltd. (Shanghai, China), and the sequences used in this experiment are listed in Table 1.

#### 2.5.4. Transcriptome Sequencing

Qualified RNA samples were subjected to mRNA enrichment using Oligo(dT) beads, followed by fragmentation and cDNA library construction. Sequencing was performed on an Illumina NovaSeq 6000 platform. Raw data were quality-controlled and filtered before alignment to the reference genome using HISAT2 v2.2.1. Gene expression levels were calculated based on FPKM values. Differentially expressed genes (DEGs) were identified using thresholds of FDR < 0.05 and |log_2_FC| > 1. Functional annotation was performed through GO and KEGG enrichment analyses (q < 0.05).

### 2.6. In Vivo Toxicity Assessment

After one week of acclimation, healthy and vigorous juvenile crabs were selected and randomly allocated into eight groups (each group had 2 replicates, with each replicate consisting of a single tank containing 20 juvenile crabs). Using a micro-syringe (100 μL), the crabs were injected in the second pereiopod with T-BHP at doses of 0, 50, 100, 150, 200, 250, 300, or 350 mg·kg^−1^ body weight; the control group received an injection of PBS. Mortality in each group was recorded at 24, 48, 72, and 96 h post-injection. The 96 h median lethal concentration (96 h-LD_50_) of T-BHP for the crabs was calculated. Deceased crabs were promptly removed during the trial, and neither water exchange nor feeding was conducted to avoid additional stress. All other husbandry practices followed the procedures described in Section 2.1.

Subsequently, a sublethal challenge was conducted using 176.88 mg·kg^−1^ T-BHP, equivalent to 60% of the 96 h-LD_50_, for a duration of 96 h. Biochemical parameters were measured at 24, 48, 72, and 96 h to determine the optimal time point for inducing oxidative stress in vivo, which was then used for subsequent in vivo experiments. Specifically, crabs were injected in the second pereiopod with either PBS (control group) or 176.88 mg·kg^−1^ T-BHP (each group had 2 replicates, with each replicate consisting of a single tank containing 20 juvenile crabs). At each time point (24, 48, 72, 96 h), five crabs were randomly sampled from each group. and their hepatopancreas tissues were collected for biochemical analysis.

#### Tissue Biochemical Analysis

Tissue homogenates were prepared in physiological saline at a weight: volume ratio of 1:9. After centrifugation, supernatants were collected for determination of ACP, AKP, GPT, GOT (510 nm), CAT, T-AOC, GSH (405 nm), T-SOD (450 nm), and TP (595 nm) levels according to manufacturer’s instructions. Protein concentrations in the samples were determined using the Bradford method [31] (Bradford, 1976), and the enzyme activity units per milligram of protein were calculated accordingly.

### 2.7. Validation of Dietary Supplementation Efficacy

After one week of acclimation, healthy juvenile crabs were selected and divided into seven groups (each group had 3 replicates, with each replicate consisting of a single tank containing 20 juvenile crabs). The blank control group and the oxidative stress model group were fed the basal diet. The allicin supplementation groups received the basal diet supplemented with 75, 150, 300, or 500 mg·kg^−1^ allicin. Additionally, the group receiving the highest dose (500 mg·kg^−1^) served as the high-dose allicin control group. After 35 days of feeding, crabs in the model group and the allicin supplementation groups were injected with 176.88 mg·kg^−1^ T-BHP, whereas those in the blank control and high-dose allicin control groups received an equivalent volume of PBS. All other husbandry practices followed the procedures described in Section 2.1. Based on the optimal stress time point determined in Section 2.6, nine crabs were randomly sampled from each group, and their hepatopancreas tissues were collected for biochemical analysis and RT-PCR, following the methodologies described in Sections Tissue Biochemical Analysis and 2.5.3, respectively.

#### Growth Performance Evaluation

At the conclusion of the feeding trial, the following growth performance indices were calculated for each group:

Weight gain rate (WGR, %) = (Final weight − Initial weight)/Initial weight × 100%

Specific growth rate (SGR, %·d^−1^) = (lnFinal weight − lnInitial weight)/Feeding days × 100%

Feed conversion ratio (FCR) = Total feed consumption/(Final weight − Initial weight)

Hepatosomatic index (HSI, %) = Hepatopancreas weight/Final weight × 100%

### 2.8. Data Processing and Statistical Analysis

All data are presented as the mean ± standard error of the mean (SEM). When the assumptions of normal distribution (Shapiro–Wilk test) and homogeneity of variance (Levene’s test) were satisfied, one-way analysis of variance (ANOVA) was performed to assess the significance of differences among treatment groups, followed by post hoc multiple comparisons using Tukey’s Honestly Significant Difference (HSD) test. Two-way ANOVA (T-BHP × Time) was employed to examine the independent and interactive effects of T-BHP exposure concentration and duration. Following a simple effects analysis, Tukey’s HSD test was applied for multiple comparisons within each dimension. If the data did not meet the assumptions of normality or homogeneity of variance, the non-parametric Kruskal–Wallis test was used instead, followed by Mann–Whitney U post hoc tests with Bonferroni correction for multiple comparisons. A probability value of *p* < 0.05 was considered statistically significant. All statistical analyses were performed using SPSS software (version 27.0.1), and data visualization was conducted using GraphPad Prism (version 9.5).

## 3. Results

### 3.1. Establishment of an In Vitro Oxidative Stress Model Induced by T-BHP and Determination of the Optimal Allicin Concentration

**Figure 1:**
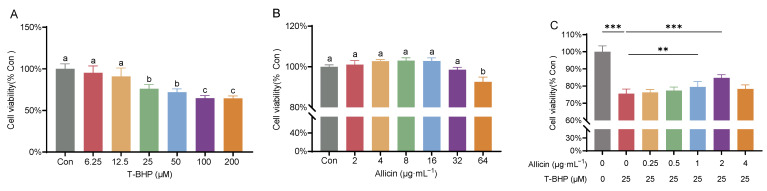
Viability of hepatopancreatic cells from Chinese mitten crabs following exposure to different concentrations of T-BHP (**A**), allicin (**B**), and allicin intervention under T-BHP exposure (**C**). Different lowercase letters indicate significant differences among treatment groups (*p* < 0.05). “**” and “***” denote significant differences between the two treatment groups (*p* < 0.01 or *p* < 0.001, respectively).

In this experiment, hepatopancreatic cells were exposed to varying concentrations of T-BHP for 4 h, and cell viability was assessed using the CCK-8 assay to determine the optimal working concentration for establishing the oxidative stress model. The results (Figure 1A) demonstrated that 25–200 μM T-BHP significantly inhibited the viability of hepatopancreatic cells in a dose-dependent manner (*p* < 0.05). Exposure to 25 μM T-BHP for 4 h resulted in a cell viability of 75.9%; therefore, this concentration was selected for subsequent model establishment to ensure the feasibility of further cellular experiments.

As shown in Figure 1B, hepatopancreatic cell viability initially increased with rising allicin concentrations, peaking at 8 μg·mL^−1^, although this increase was not statistically significant (*p* > 0.05). However, further increases in allicin concentration led to a decline in cell viability, with a significant cytotoxic effect observed at 64 μg·mL^−1^ (*p* < 0.05).

Based on the aforementioned findings, we utilized the T-BHP-induced cellular oxidative stress model to further determine the optimal intervention concentration of allicin. The results, depicted in Figure 1C, revealed that pretreatment with 1–2 μg·mL^−1^ allicin significantly attenuated the T-BHP-induced decrease in cell viability (*p* < 0.05) compared to the model group. Among these concentrations, 2 μg·mL^−1^ allicin exhibited the most pronounced protective effect. Consequently, 2 μg·mL^−1^ was selected for all subsequent in vitro investigations.

### 3.2. Allicin Ameliorates T-BHP-Induced Oxidative Damage in Hepatopancreatic Cells

#### 3.2.1. Effects on Biochemical Parameters

**Figure 2:**
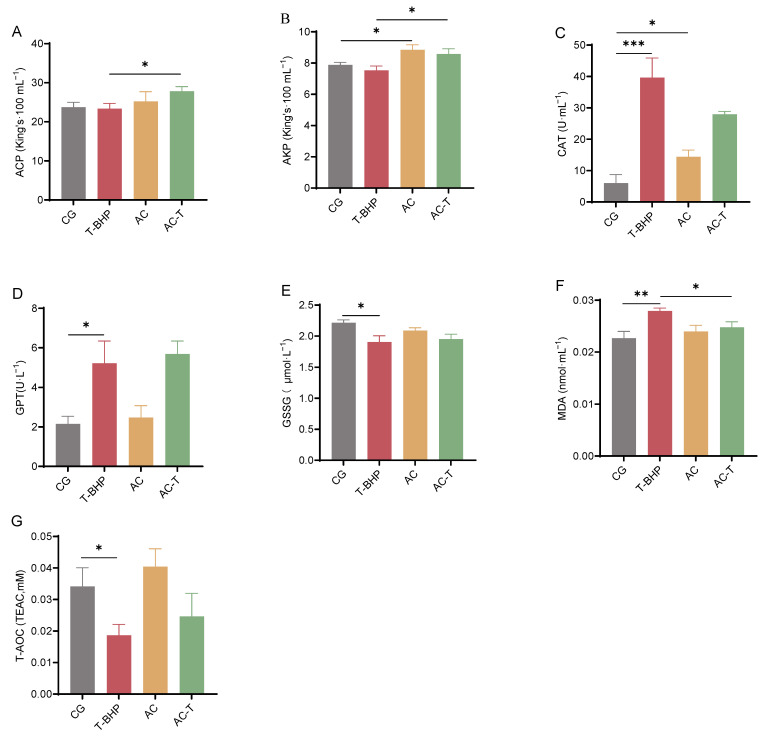
Effects of T-BHP exposure and allicin intervention on biochemical parameters in the culture supernatant of hepatopancreatic cells from Chinese mitten crabs. (**A**,**B**) Immune-related parameters: ACP, AKP. (**C**) Liver injury parameter: GPT. (**D**–**G**) Antioxidant-related parameters: CAT, T-AOC, GSSG, MDA. “*”, “**” and “***” denote significant differences between the two treatment groups (*p* < 0.05 or *p* < 0.01 or *p* < 0.001, respectively).

Compared to the blank control group (CG), exposure to 25 μM T-BHP for 4 h significantly increased the levels of GPT, CAT, and MDA (*p* < 0.05, Figure 2C,D,F) while significantly decreasing GSSG (*p* < 0.05, Figure 2E) in the hepatopancreatic cell culture supernatant. Compared to the model group (T-BHP), Pretreatment with 2 μg·mL^−1^ allicin for 24 h significantly enhanced the activity of AKP (*p* < 0.05, Figure 2B), and suppressed the T-BHP-induced increases in CAT and MDA (*p* < 0.05, Figure 2D,F), although the difference is not significant.

#### 3.2.2. Effects on Gene Expression

**Figure 3:**
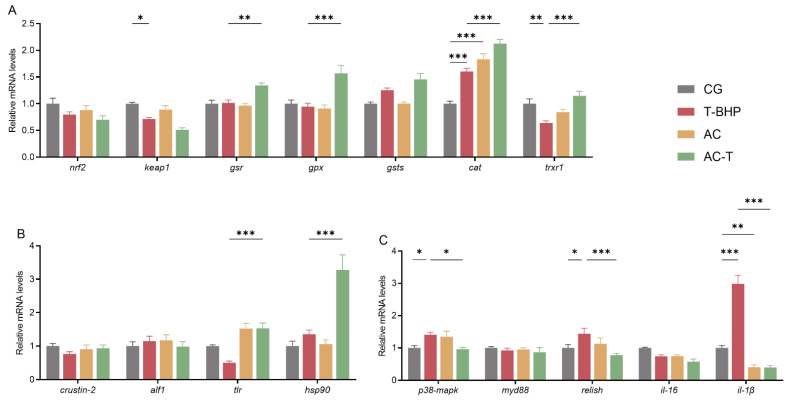
Effects of T-BHP exposure and allicin intervention on gene expression in hepatopancreatic cells of Chinese mitten crabs. (**A**) Antioxidant-related genes: *nrf2*, *keap1*, *gsr*, *gpx*, *gsts*, *cat*, *trxr1*. (**B**) Immune-related genes: *crustin-2*, *alf1*, *tlr*, *hsp90*. (**C**) Inflammation-related genes: *p38-mapk*, *myd88*, *relish*, *il-16*, *il-1β*. “*”, “**” and “***” denote significant differences between the two treatment groups (*p* < 0.05 or *p* < 0.01 or *p* < 0.001, respectively).

As illustrated in Figure 3, compared to the CG group, exposure to 25 μM T-BHP for 4 h significantly downregulated the expression levels of *keap1* and *trxr1* while significantly upregulating the expression of *cat*, *p38-mapk*, *relish* and *il-1β* (*p* < 0.05) in hepatopancreatic cells. In the AC-T group, compared to the T-BHP group, the expression levels of *p38-mapk*, *relish*, and *il-1β* were significantly downregulated, whereas the expression of *gsr*, *gpx*, *cat*, *trxr1*, *tlr*, and *hsp90* was significantly upregulated (*p* < 0.05).

#### 3.2.3. Ultrastructural Observations

**Figure 4:**
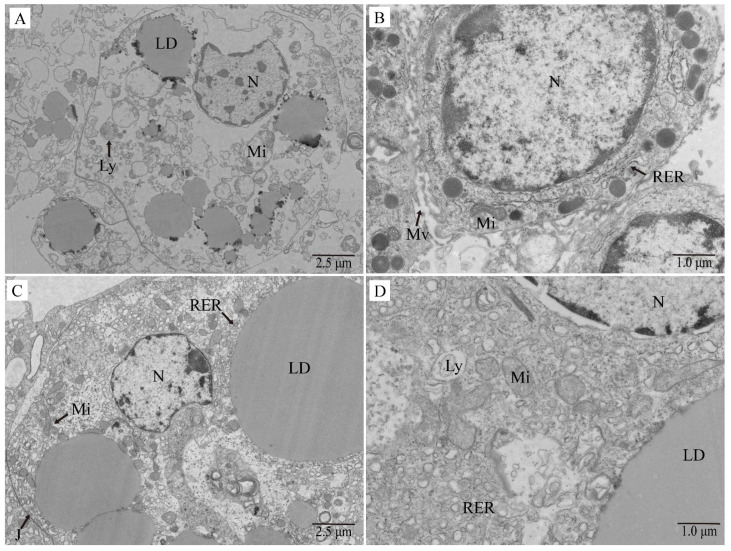
Ultrastructure of hepatopancreatic cells from Chinese mitten crabs under T-BHP exposure and allicin intervention. (**A**,**B**) Cells from the T-BHP group; (**C**,**D**) Cells from the AC-T group. N: Nucleus; LD: Lipid droplet; Mi: Mitochondria; RER: Rough endoplasmic reticulum; J: Cell–cell junction; Ly: Lysosome; Mv: Microvilli.

Electron microscopy observations revealed that in the T-BHP-challenged group, most intracellular organelles such as mitochondria and the endoplasmic reticulum were swollen, lysed, and vacuolated; lipid droplets were ruptured; cytoplasmic loss was evident and lysosomes appeared, indicating the onset of cell necrosis (Figure 4A). In cells that had not undergone necrosis, the number of mitochondria was drastically reduced with aberrant morphology; the endoplasmic reticulum was swollen, deformed, and disorganized, containing numerous vacuoles; most microvilli were fractured and shed (Figure 4B). In contrast, cells from the allicin-protected group (AC-T) exhibited intact mitochondrial structures with regular, mostly round or oval shapes; plump lipid droplets; tight cell–cell junctions with clear and intact boundaries; and neatly arranged rough endoplasmic reticulum densely studded with ribosomes (Figure 4C,D).

#### 3.2.4. Transcriptomic Analysis

**Figure 5:**
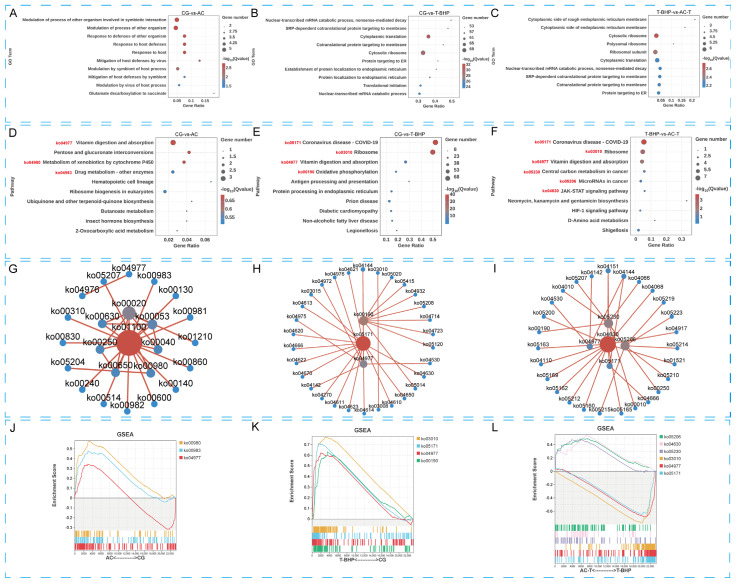
Transcriptomic analysis of hepatopancreatic cells from Chinese mitten crabs under T-BHP exposure and allicin intervention. (**A**–**C**) Top 10 GO enrichment bubble plots for CG vs. AC, CG vs. T-BHP, and T-BHP vs. AC-T, respectively. (**D**–**F**) Top 10 KEGG enrichment plots for CG vs. AC, CG vs. T-BHP, and T-BHP vs. AC-T, respectively. (**G**–**I**) KEGG pathway network diagrams for CG vs. AC, CG vs. T-BHP, and T-BHP vs. AC-T, respectively. (**J**–**L**) GSEA analysis plots for key KEGG pathways in CG vs. AC, CG vs. T-BHP, and T-BHP vs. AC-T, respectively.

To further explore the potential antioxidant mechanisms of allicin, we performed transcriptomic analysis on hepatopancreatic cells, with the results summarized in Figure 5. GO and KEGG enrichment analyses indicated significant differences in oxidation-, immunity-, and inflammation-related pathways among the groups. In the GO analysis, compared to the CG group, differentially expressed genes (DEGs) in the T-BHP group were significantly enriched in biological processes and cellular components such as nuclear-transcribed mRNA catabolic process and co-translational protein targeting to membrane (Figure 5B), suggesting that cells under oxidative stress respond to damage by regulating mRNA metabolism and protein subcellular localization. In contrast, DEGs in the AC-T group were enriched in terms like rough endoplasmic reticulum membrane and polysome (Figure 5C), indicating that allicin pretreatment may counteract oxidative damage by modulating endoplasmic reticulum and ribosome functions. Furthermore, DEGs in the AC group were significantly enriched in biological processes including response to host defenses and modulation by virus and symbiont of host defenses (Figure 5A), hinting that allicin intervention might alleviate immune and inflammatory responses by regulating host-microbe interactions.

In the KEGG pathway analysis, the oxidative phosphorylation pathway was significantly upregulated in the T-BHP group, reflecting a sharp increase in oxidative stress levels. Concurrently, immune-inflammatory pathways such as Coronavirus disease—COVID-19 and Antigen processing and presentation were enriched (Figure 5E,H,K), suggesting that oxidative stress was accompanied by immune activation. Compared to the T-BHP group, the AC-T group showed ameliorated expression patterns of DEGs related to immune pathways like the JAK-STAT signaling pathway and the oxidative phosphorylation pathway. Furthermore, core pathways including Coronavirus disease—COVID-19 and Ribosome were enriched in the AC-T group (Figure 5F,I,L), indicating that allicin effectively alleviates T-BHP-induced oxidative and immune dysregulation by modulating oxidative metabolism, immune-inflammatory signaling (e.g., JAK-STAT), and protein synthesis pathways. Additionally, the differential enrichment in metabolic pathways such as Vitamin digestion and absorption (Figure 5D–I) provides supplementary evidence for the multi-faceted regulatory role of allicin.

### 3.3. Toxic Effects of T-BHP Exposure in Juvenile Crabs

**Figure 6:**
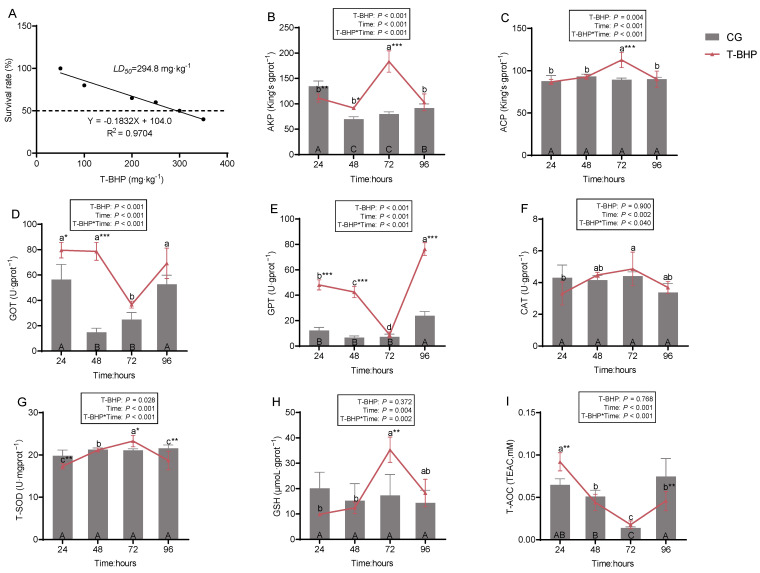
Toxic effects of T-BHP exposure on juvenile crabs. (**A**) The 96 h-LD_50_ value derived from linear regression of crab survival rates after 96 h exposure to different doses of T-BHP. (**B**,**C**) Immune-related parameters: AKP, ACP. (**D**,**E**) Liver injury parameters: GPT, GOT. (**F**–**I**) Antioxidant-related parameters: CAT, T-SOD, GSH, T-AOC. The boxes show the *p*-values for the main and interaction effects of T-BHP exposure treatment and exposure duration. Different uppercase letters indicate significant differences (*p* < 0.05) among different time points within the CG group, different lowercase letters indicate significant differences (*p* < 0.05) among different time points within the T-BHP group, and “*”, “**” and “***” denote significant differences between the CG and T-BHP groups at the same time point (*p* < 0.05 or *p* < 0.01 or *p* < 0.001, respectively).

The 96 h acute toxicity test determined that the 96 h-LD_50_ of T-BHP in juvenile crabs was 294.8 mg·kg^−1^ (Figure 6A).

After challenge with 60% of the 96 h-LD_50_ (176.88 mg·kg^−1^), dynamic changes in hepatopancreatic biochemical parameters were monitored. As shown in Figure 6, The Two-way ANOVA analysis showed that T-BHP exposure treatment had no significant effect on the levels of CAT, GSH, and T-AOC (Figure 6F,H,I). However, the main effect of exposure time alone, as well as the interaction between exposure treatment and exposure time, were significant (*p* < 0.05). For all other indicators, significant effects were observed under the individual and interactive influences of both T-BHP exposure treatment and exposure time (*p* < 0.05). during 24–96 h post-T-BHP exposure, AKP, ACP, CAT, T-SOD, and GSH levels initially increased and then decreased (Figure 6B,C,F–H), whereas GOT, GPT, and T-AOC first decreased and then increased (Figure 6D,E,I), all parameters reached extremes at 72 h. At the 24 h time point, most parameters (except ACP, CAT, GSH) showed significant differences between the T-BHP and CG groups (*p* < 0.05).

### 3.4. Dietary Allicin Promotes Growth, Antioxidant Capacity, and Immune Function in Juvenile Crabs

#### 3.4.1. Effects on Growth Performance

**Figure 7:**
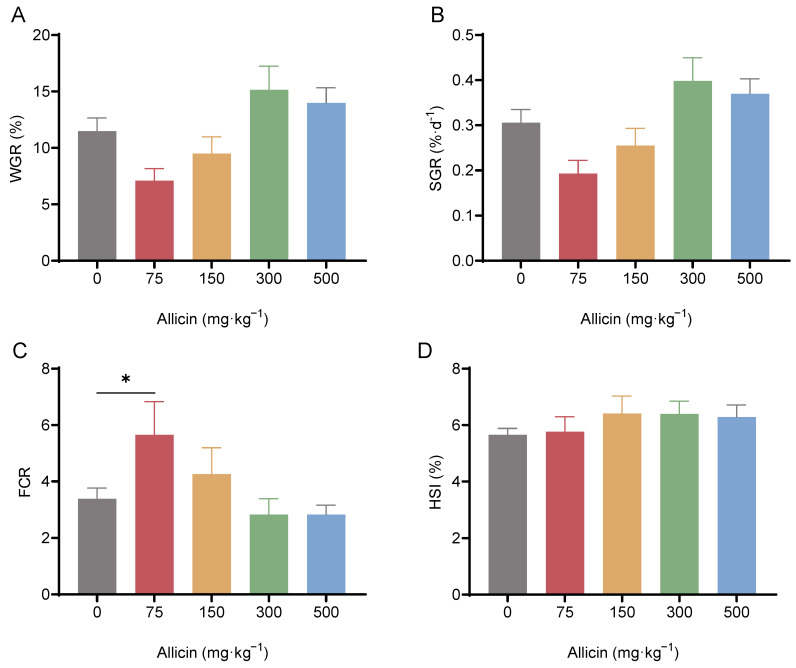
Effects of dietary supplementation with different doses of allicin on weight gain rate, WGR (**A**), specific growth rate, SGR (**B**), feed conversion ratio, FCR (**C**), and hepatosomatic index, HSI (**D**) of Chinese mitten crabs. “*” denotes significant differences between the two treatment groups (*p* < 0.05).

As shown in Figure 7, compared to the blank control group, dietary supplementation with 300 and 500 mg·kg^−1^ allicin improved weight gain rate (WGR) and specific growth rate (SGR), and reduced feed conversion ratio (FCR) though not significantly; in contrast, 75 and 150 mg·kg^−1^ groups decreased WGR and SGR, and 75 mg·kg^−1^ groups significantly increased FCR (*p* < 0.05, Figure 7A–C). Allicin supplementation increased hepatosomatic index (HSI) across all groups without significant differences (Figure 7D). Overall, growth performance showed an initial increase followed by a decrease with rising allicin doses, with the 300 and 500 mg·kg^−1^ group performing best.

#### 3.4.2. Effects on Biochemical Parameters

**Figure 8:**
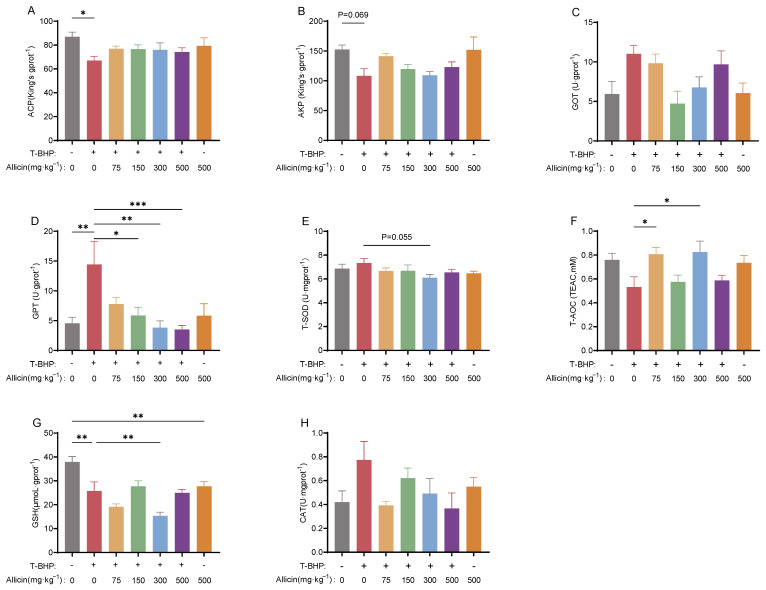
Effects of different dietary allicin supplementation levels on hepatopancreatic biochemical parameters in juvenile crabs under T-BHP exposure. (**A**,**B**) Immune-related parameters: ACP, AKP. (**C**,**D**) Liver injury parameters: GOT, GPT. (**E**–**H**) Antioxidant-related parameters: T-SOD, T-AOC, GSH, CAT. “*”, “**” and “***” denote significant differences between the two treatment groups (*p* < 0.05 or *p* < 0.01 or *p* < 0.001, respectively).

Compared to the blank control, T-BHP exposure significantly reduced ACP activity (*p* < 0.05, Figure 8A), and increased GPT activity (*p* < 0.05, Figure 8D), indicating hepatotoxicity and immunosuppression. Concurrently, GSH level were significantly decreased (*p* < 0.05, Figure 8G), suggesting impaired antioxidant capacity. Compared to the model group, all allicin supplementation groups enhanced ACP, AKP activities and T-AOC (Figure 8A,B,F), and reduced GOT, GPT, T-SOD, and CAT activities (Figure 8C–E,H) though not all show significant differences. The 300 mg·kg^−1^ group exhibited the most pronounced effects, significantly reversing T-BHP-induced abnormalities in GPT, T-AOC, and GSH (*p* < 0.05, Figure 8D,F,G).

#### 3.4.3. Effects on Gene Expression

**Figure 9:**
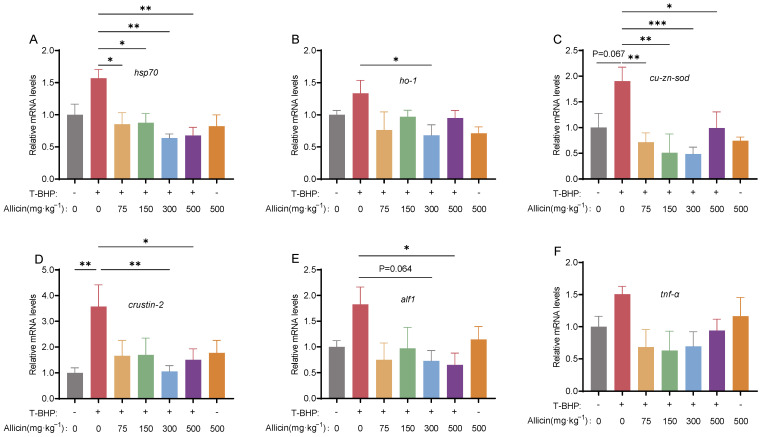
Effects of different dietary allicin supplementation levels on hepatopancreatic gene expression in juvenile crabs under T-BHP exposure. (**A**–**C**) Antioxidant-related genes: *hsp70*, *ho-1*, *cu-zn-sod*. (**D**–**F**) Immune-related genes: *crustin-2*, *alf1*, *tnf-α*. “*”, “**” and “***” denote significant differences between the two treatment groups (*p* < 0.05 or *p* < 0.01 or *p* < 0.001, respectively).

As shown in Figure 9, compared to the CG group, the T-BHP group showed upregulation of oxidative stress-related genes (*hsp70*, *ho-1*, *cu-zn-sod*) and immune-inflammatory genes (*crustin-2*, *alf1*, *tnf-α*), with significant difference in *crustin-2* (*p* < 0.05 Figure 9D). Allicin intervention (75–500 mg·kg^−1^) suppressed T-BHP-induced gene upregulation, with the 300 mg·kg^−1^ group exhibiting the most significant inhibition, showing statistical differences in most indicators (except *tnf-α*) compared to the model group (*p* < 0.05).

## 4. Discussion

T-BHP, an organic peroxide [25], has been validated for its ability to induce oxidative stress through ROS accumulation and disruption of redox homeostasis in various biological models [24,32], and is widely used in both in vivo and in vitro oxidative damage studies [24]. Our results demonstrate that 4 h exposure to 25 μM T-BHP significantly reduced hepatopancreatic cell viability and triggered characteristic oxidative damage markers: substantial generation of MDA, along with decreased GSSG levels. These alterations align with observations in mammalian cells and tissues [33,34], confirming T-BHP as a reliable oxidative stress inducer capable of provoking robust oxidative damage in crustacean cells. In vivo experiments further substantiated this finding, showing acute increases in transaminase (GOT, GPT) activities and decreased ACP and GSH levels in hepatopancreas, clearly indicating substantial tissue damage. The successful establishment of this model provides a foundation for precisely investigating the protective mechanisms of allicin at cellular level.

In this study, pretreatment with 2 μg·mL^−1^ allicin significantly ameliorated T-BHP-induced cell viability reduction, enhanced AKP activity, and concurrently decreased MDA content, demonstrating remarkable antioxidant potential. Similarly, curcumin pretreatment has been reported to maintain tilapia hepatocyte viability and reduce MDA levels under H_2_O_2_ stress [35]. In feeding trials, dietary allicin (75–500 mg·kg^−1^) effectively reversed T-BHP-induced decreases in T-AOC activity, and elevations in GPT level, consistent with the hepatoprotective effects of ICA on Chinese mitten crabs reported by Zheng et al. [12]. Their study demonstrated that dietary supplementation with 100 mg·kg^−1^ ICA significantly suppressed LPS-induced increases in MDA, GOT, and GPT levels in crab hepatopancreas, further supporting the potential of allicin as an effective feed additive for mitigating oxidative stress-induced hepatotoxicity.

*Nrf2*, an oxidative stress-sensitive transcription factor [36], remains inactive through binding with *keap1* under basal conditions [37]. Upon oxidative challenge, *nrf2* activates and translocates to the nucleus, initiating transcription of downstream antioxidant enzymes (e.g., GSH, GPx, GSTs, HO-1) [31,32]. Studies have shown that curcumin treatment ameliorates oxidative stress and inflammation in nephrectomized rats by reducing *keap1* levels while elevating *nrf2* and *ho-1* expression [38]. In grass carp under heat stress, AS-IV potentially enhances hepatocyte antioxidant capacity via the Keap1-Nrf2 pathway by modulating expression of *keap1a*, *nrf2*, *gsh-px*, *ho-1*, and *il-6* [39]. In our study, T-BHP activated the Nrf2-Keap1 pathway, evidenced by *keap1* downregulation and enhanced transcription of antioxidant genes including *gsts* and *cat*. Allicin pretreatment further modulated this pathway, not only promoting keap1 downregulation but also significantly upregulating *gpx* and *trxr1* expression while reducing MDA content and restoring antioxidant enzyme system balance. The upregulation of GPX and TRXR1, key enzymes for peroxide scavenging and intracellular redox maintenance [40,41,42], directly enhanced cellular antioxidant reserves. These findings suggest that allicin may facilitate Nrf2 nuclear translocation and transcriptional activity, potentially initiating Antioxidant Response Element (ARE)-driven gene expression programs [38,43], consistent with the antioxidant mechanisms of other natural extracts like curcumin and AS-IV.

*p38-mapk*, a stress-sensitive kinase activated by ROS [1], phosphorylates and activates downstream transcription factor *nf-κb*, driving synthesis of pro-inflammatory cytokines such as IL-16 and IL-1β [44,45,46]. The interconnection between oxidative stress and inflammatory response constitutes an important mechanism underlying tissue damage. Our study reveals that the protective effects of allicin extend beyond antioxidation to include anti-inflammatory actions. T-BHP exposure significantly activated *p38-mapk* and sharply upregulated *il-1β* gene expression, indicating oxidative damage triggered classical inflammatory signaling cascades. Allicin pretreatment significantly suppressed key pathway genes including *p38-mapk* and *relish*, and downregulated *il-1β* levels, suggesting its anti-inflammatory effect may involve inhibition of *p38-mapk* phosphorylation, thereby blocking NF-κB pathway activation. This mechanism aligns with reports by Che et al. [18] that allicin exerts anti-inflammatory effects in bovine mammary epithelial cells via the TLR4/NF-κB pathway, suggesting conserved anti-inflammatory pathway inhibition across species. Transcriptomic analysis further confirmed that allicin reversed T-BHP-induced enrichment of “COVID-19” pathway, mitigating nonspecific inflammatory responses by downregulating inflammation-related genes including *ace* and *vwf* [47,48]. Through concurrently enhancing antioxidant capacity and suppressing inflammatory activation, allicin achieves dual containment of oxidative damage.

Ultrastructural observations revealed that T-BHP exposure induced mitochondrial swelling and rupture, endoplasmic reticulum vacuolization, lipid droplet rupture, and impaired cell junctions in hepatopancreatic cells, while allicin pretreatment significantly alleviated these organellar structural damages. Mechanistically, T-BHP group showed significant upregulation of “oxidative phosphorylation” pathway, with impaired mitochondrial complex subunit function leading to energy metabolism disruption. Allicin restored mitochondrial functional stability by modulating this pathway while upregulating *hsp90* expression to enhance chaperone-mediated protein quality control [49,50], maintaining proteostasis under stress conditions. This finding aligns with the known mitochondrial protective [51] and proteostasis maintenance [52] effects of allicin.

Enrichment of “vitamin digestion and absorption” pathway represents an important finding. Alliin-treated group showed significant upregulation of vitamin C transporter SLC23A1, and since vitamin C serves as a key antioxidant cofactor promoting glutathione regeneration, this further enhances intracellular endogenous antioxidant capacity [53,54]. Additionally, allicin may mimic mild stress through hormesis effects [55], activating cellular defense preconditioning mechanisms, as evidenced by increased AKP and CAT activities in allicin control group and enrichment of host defense regulation pathways, suggesting its protection extends beyond direct ROS scavenging to involve systemic enhancement in organismal stress resistance.

Dietary allicin has been reported to positively influence growth, immunity, and antioxidant capacity in large yellow croaker (*Larimichthys Crocea*) larvae [56]. After 30-day feeding with 0.01% dietary allicin, survival rate and specific growth rate were significantly higher than controls, showing an initial increase followed by decrease within certain dosage range; concurrently, allicin enhanced T-AOC, CAT, and NOS activities while reducing *il-1β* and *il-6* transcript levels [50]. In our feeding trial, higher dietary allicin doses (300 and 500 mg·kg^−1^) improved weight gain rate and specific growth rate while reducing feed conversion ratio, though some growth parameters did not reach statistical significance. Notably, growth performance showed a declining trend at 500 mg·kg^−1^. Dietary allicin supplementation (75–500 mg·kg^−1^) ameliorated T-BHP-induced hepatopancreatic oxidative stress and liver damage, with the 300 mg·kg^−1^ group exhibiting optimal efficacy in reversing abnormalities in antioxidant enzyme activities, immune and inflammatory factors, consistent with effective dosage ranges reported for allicin in aquatic species including large yellow croaker [56], tilapia [20], and white-leg shrimp [22], supporting its application potential in crustacean nutrition and health management.

## 5. Conclusions

In summary, allicin effectively alleviates T-BHP-induced hepatopancreatic oxidative damage and inflammatory responses in Chinese mitten crabs. The protective mechanisms involve synergistic actions across multiple pathways: at the cellular level, allicin enhances antioxidant defense likely through activating the Nrf2 signaling pathway and mitigates inflammatory response possibly by suppressing the p38-MAPK/NF-κB pathway. Transcriptomic analyses further reveal that its regulation of proteostasis, mitochondrial function, and vitamin metabolism constitutes important complementary mechanisms. Feeding trials identify 300 mg·kg^−1^ as the optimal dietary supplementation dose, significantly improving growth performance and hepatopancreatic health. This study provides solid theoretical foundation for developing allicin as a green and efficient antioxidant feed additive in aquaculture. It should be acknowledged that the mechanistic inferences regarding pathway activation (Nrf2, p38-MAPK/NF-κB) are primarily based on gene expression profiles and biochemical endpoints. Direct protein-level evidence (e.g., Nrf2 nuclear translocation) and functional validation using specific pathway inhibitors or genetic approaches were not performed in this study, which precludes definitive causal conclusions. Furthermore, the sample size in some analyses may limit the generalizability of the findings. Future studies incorporating pharmacological inhibitors and protein assays are warranted to establish causality.

## Figures and Tables

**Table 1:** Primers sequences for RT-PCR.

Gene	Sequence (5′-3′)	Sources
*nrf2*	F: ACCACAGAAATGAACCAAACCAC	XP_027208873.1
	R: GTCAGGACTAAGGGAAGACACTG	
*keap1*	F: CTTGCTGTACCTTTCTTGAGCAG	XP_027210665.1
	R: CTGCAAAAACTCCTCCTCCAATG	
*gsr*	F: GGCGAAGGAACACTGCATCA	XM_050838252.1
	R: CCAGGGAGATATACGACGCC	
*gpx*	F: GACTACACCCCAGTGTGCACCA	FJ617305.1
	R: TGATCCAGCCATTGTGATCCTC	
*gsts*	F: AAGAGTCGAGTATGAGGACAAGC	XM_050837187.1
	R: AGCGTCTGAGTTATCTTCACGTT	
*cat*	F: CAAGACAGGGCCATCAATAACTTC	Accession: GU361618.1
	R: TCACTGCATGGACAGTTGAAAGA	
*trxr1*	F: CAGGGAGAAGAAAGTGGACTACC	XM_050838252.1
	R: ATATCGTCTGAGGTGATGCAGTG	
*crustin-2*	F: GCCCACCTCCCAAACCTAT	XM_050862131.1
	R: GCAAGCGTCACAGCAGCACT	
*alf1*	F: GCTGGCTGGACCGGATTATT	XM_050871541.1
	R: ATCACACGGGTGTTGCAGAT	
*tlr*	F: AGCTTGCCGATTCACACTCA	XM_050878071.1
	R: CACAGCTCTTCCTCCGTCAG	
*hsp90*	F: TCACCAACGACTGGGAGGAT	XM_050873093
	R: CAGGAAGAGGAGTGCCCTGA	
*p38-mapk*	F: CACTCATGGGTGCTGACCTC	XM_050867549.1
	R: TACTTGAGGCCTCGCAACAC	
*myd88*	F: GCCATCGCAGTCGCCAAGTT	XM_050877733.1
	R: GGCATCCTGTTCATCCAGTTCTGAC	
*relish*	F: TCTCCCTACTCTGACCATTCC	XM_050843537.1
	R: TTCCCACCATCTCACTCTTGT	
*il-16*	F: AGAGGTTGTTCTTGTGCTGTCC	XM_050877878.1
	R: ACGAGGGTAATGGTGAATGGAG	
*il-1β*	F: ATCAGCTGAAGTCCATCAGCCAGCA	[30]
	R: TGCATGTCCGTGCTGATGAACCAGT	
*hsp70*	F: TCCCAGCGTACTTTAACGATTCA	XM_050870185.1
	R: TCGTAGAACATTTAGTCCCGCAA	
*ho-1*	F: TCGACCTCAACCTTGAAGCA	XM_050878156.1
	R: TGCATCACTGACACCGTGAA	
*cu-zn-sod*	F: ATGAGTAAGACCTTTGCCTA	XM_050882595.1
	R: TCCGTCAGTCCATAGATAAC	
*tnf-α*	F: GTGGACATCTGGTCAGTGGG	XM_050867549.1
	R: GGCTCATCTGAGGGATCTGC	
*s27*	F: GGTCGATGACAATGGCAAGA	XM_050861302.1
	R: CCACAGTACTGGCGGTCAAA	

*nrf2*: Nuclear factor-erythroid 2–related factor 2; *keap1*: Kelch-like erythroid cell-derived protein with cap-ncollar homology-associated protein 1; *gsr*: Glutathione reductase; *gpx*: Glutathione peroxidase; *gsts*: Glutathione S-transferase sigma; *cat*: Catalase; *trxr1*: Thioredoxin reductase 1; *crustin-2*: Antimicrobial Peptide; *alf1*: Anti-lipopolysaccharide factor 1; *tlr*: Toll-like receptors; *hsp90*: Heat Shock Protein 90; *p38-mapk*: p38 Mitogen-Activated Protein Kinase; *myd88*: Myeloid Differentiation Primary Response 88; *relish*: The NF-κB factor; *il-16*: Interleukin-16; *il-1β*: Interleukin-1 beta; *hsp70*: Heat Shock Protein 70; *ho-1*: Heme oxygenase-1; *cu-zn-sod*: Cu,Zn-Superoxide Dismutase; *tnf-α*: Tumor necrosis factor-alpha; *s27*: ribosomal protein S27-like.

## Data Availability

The raw data supporting the conclusions of this article will be made available by the authors on request.

## Abbreviations

The following abbreviations are used in this manuscript:

ICA	Icariin
LPS	lipopolysaccharide
T-BHP	Tert-Butyl Hydroperoxide
*il-1β*	Interleukin-1 beta
*il-16*	Interleukin-16
*ho-1*	Heme oxygenase-1
MDA	Malondialdehyde
*nrf2*	Nuclear factor-erythroid 2–related factor 2
*tnf-α*	Tumor necrosis factor-alpha
PBS	Phosphate-buffered saline
ACP	Acid phosphatase
AKP	Alkaline phosphatase
GPT	Glutamic pyruvic transaminase
CAT	Catalase
T-AOC	Total antioxidant capacity
GSSG	Oxidized glutathione
*trxr1*	Thioredoxin reductase 1
*gsr*	Glutathione reductase
*gpx*	Glutathione peroxidase
*gsts*	Glutathione S-transferase sigma
*crustin-2*	Antimicrobial Peptide
*alf1*	Anti-lipopolysaccharide factor 1
*tlr*	Toll-like receptors
*hsp90*	Heat Shock Protein 90
*p38-mapk*	p38 Mitogen-Activated Protein Kinase
*myd88*	Myeloid Differentiation Primary Response 88
*relish*	The NF-κB factor
GOT	Glutamic oxaloacetic transaminase
T-SOD	Total Superoxide Dismutase
GSH	Reduced glutathione
*hsp70*	Heat Shock Protein 70
*cu-zn-sod*	Cu,Zn-Superoxide Dismutase
*s27*	ribosomal protein S27-like
*keap1*	Kelch-like erythroid cell-derived protein with cap-ncollar homology-associated protein 1

## References

[1] Afzal S., Abdul Manap A.S., Attiq A., Albokhadaim I., Kandeel M., Alhojaily S.M. (2023). From Imbalance to Impairment: The Central Role of Reactive Oxygen Species in Oxidative Stress-Induced Disorders and Therapeutic Exploration. Front. Pharmacol..

[2] Fisheries and Fishery Administration Bureau of the Ministry of Agriculture and Rural Affairs (2025). Fisheries Statistical Yearbook of China 2025.

[3] Menon S.V., Kumar A., Middha S.K., Paital B., Mathur S., Johnson R., Kademan A., Usha T., Hemavathi K.N., Dayal S. (2023). Water Physicochemical Factors and Oxidative Stress Physiology in Fish, a Review. Front. Environ. Sci..

[4] Frías-Espericueta M.G., Bautista-Covarrubias J.C., Osuna-Martínez C.C., Delgado-Alvarez C., Bojórquez C., Aguilar-Juárez M., Roos-Muñoz S., Osuna-López I., Páez-Osuna F. (2022). Metals and Oxidative Stress in Aquatic Decapod Crustaceans: A Review with Special Reference to Shrimp and Crabs. Aquat. Toxicol..

[5] Lee J.-W., Jo A.-H., Lee D.-C., Choi C.Y., Kang J.-C., Kim J.-H. (2023). Review of Cadmium Toxicity Effects on Fish: Oxidative Stress and Immune Responses. Environ. Res..

[6] Xu Z., Cao J., Qin X., Qiu W., Mei J., Xie J. (2021). Toxic Effects on Bioaccumulation, Hematological Parameters, Oxidative Stress, Immune Responses and Tissue Structure in Fish Exposed to Ammonia Nitrogen: A Review. Animals.

[7] Xu Z., Regenstein J.M., Xie D., Lu W., Ren X., Yuan J., Mao L. (2018). The Oxidative Stress and Antioxidant Responses of *Litopenaeus vannamei* to Low Temperature and Air Exposure. Fish Shellfish Immunol..

[8] Xu X., Liu A., Hu S., Ares I., Martínez-Larrañaga M.-R., Wang X., Martínez M., Anadón A., Martínez M.-A. (2021). Synthetic Phenolic Antioxidants: Metabolism, Hazards and Mechanism of Action. Food Chem..

[9] Li S., Xie J., Bai Y., Jiang Z., Li K., Wu C. (2023). Synthetic Phenolic Antioxidants Evoked Hepatoxicity in Grass Carp (*Ctenopharyngodon idella*) through Modulating the ROS-PI3K/mTOR/AKT Pathway: Apoptosis-Autophagy Crosstalk. Fish Shellfish Immunol..

[10] Alagawany M., Farag M.R., Abdelnour S.A., Dawood M.A.O., Elnesr S.S., Dhama K. (2021). Curcumin and Its Different Forms: A Review on Fish Nutrition. Aquaculture.

[11] Hsieh T.-J., Wang J.-C., Hu C.-Y., Li C.-T., Kuo C.-M., Hsieh S.-L. (2008). Effects of Rutin from *Toona Sinensis* on the Immune and Physiological Responses of White Shrimp (*Litopenaeus vannamei*) under *Vibrio alginolyticus* Challenge. Fish Shellfish Immunol..

[12] Zheng X., Jiang W., Zhang L., Abasubong K.P., Zhang D., Li X., Jiang G., Chi C., Liu W. (2022). Protective Effects of Dietary Icariin on Lipopolysaccharide-Induced Acute Oxidative Stress and Hepatopancreas Injury in Chinese Mitten Crab, *Eriocheir sinensis*. Comp. Biochem. Physiol. Part C Toxicol. Pharmacol..

[13] Borlinghaus J., Albrecht F., Gruhlke M.C.H., Nwachukwu I.D., Slusarenko A.J. (2014). Allicin: Chemistry and Biological Properties. Molecules.

[14] Ilić D.P., Stojanović S., Najman S., Nikolić V.D., Stanojević L.P., Tačić A., Nikolić L.B. (2015). Biological Evaluation of Synthesized Allicin and Its Transformation Products Obtained by Microwaves in Methanol: Antioxidant Activity and Effect on Cell Growth. Biotechnol. Biotechnol. Equip..

[15] Feldberg R.S., Chang S.C., Kotik A.N., Nadler M., Neuwirth Z., Sundstrom D.C., Thompson N.H. (1988). In Vitro Mechanism of Inhibition of Bacterial Cell Growth by Allicin. Antimicrob. Agents Chemother..

[16] Li X.-H., Li C.-Y., Xiang Z.-G., Hu J.-J., Lu J.-M., Tian R.-B., Jia W. (2012). Allicin Ameliorates Cardiac Hypertrophy and Fibrosis through Enhancing of Nrf2 Antioxidant Signaling Pathways. Cardiovasc. Drugs Ther..

[17] Ding G., Zhao J., Jiang D. (2016). Allicin Inhibits Oxidative Stress-Induced Mitochondrial Dysfunction and Apoptosis by Promoting PI3K/AKT and CREB/ERK Signaling in Osteoblast Cells. Exp. Ther. Med..

[18] Che H.-Y., Zhou C.-H., Lyu C.-C., Meng Y., He Y.-T., Wang H.-Q., Wu H.-Y., Zhang J.-B., Yuan B. (2023). Allicin Alleviated LPS-Induced Mastitis via the TLR4/NF-κB Signaling Pathway in Bovine Mammary Epithelial Cells. Int. J. Mol. Sci..

[19] Cai P., Zhu Q., Cao Q., Bai Y., Zou H., Gu J., Yuan Y., Liu X., Liu Z., Bian J. (2021). Quercetin and Allicin Can Alleviate the Hepatotoxicity of Lead (Pb) through the PI3K Signaling Pathway. J. Agric. Food Chem..

[20] Hamed H.S., Ismal S.M., Faggio C. (2021). Effect of Allicin on Antioxidant Defense System, and Immune Response after Carbofuran Exposure in Nile Tilapia, Oreochromis Niloticus. Comp. Biochem. Physiol. C-Toxicol. Pharmacol..

[21] Tanekhy M., Fall J. (2015). Expression of Innate Immunity Genes in Kuruma Shrimp Marsupenaeus Japonicus after in Vivo Stimulation with Garlic Extract (Allicin). Vet. Med..

[22] Huang H., Pan L., Pan S., Song M. (2018). Effects of Dietary Herbal Formulae Combined by Astragalus Polysaccharides, Chlorogenic Acid and Allicin in Different Combinations and Proportions on Growth Performance, Non-Specific Immunity, Antioxidant Status, Vibriosis Resistance and Damage Indexes Of *Litopenaeus vannamei*. Aquac. Res..

[23] Hannan M.A., Rahman M.M., Mondal M.N., Chandra D.S., Chowdhury G.A.Z.L.I.M.A., Islam M.T. (2019). Molecular Identification of *Vibrio alginolyticus* Causing Vibriosis in Shrimp and Its Herbal Remedy. Pol. J. Microbiol..

[24] Kim Y.-S., Hwang J.-W., Sung S.-H., Jeon Y.-J., Jeong J.-H., Jeon B.-T., Moon S.-H., Park P.-J. (2015). Antioxidant Activity and Protective Effect of Extract of *Celosia cristata* L. Flower on Tert-Butyl Hydroperoxide-Induced Oxidative Hepatotoxicity. Food Chem..

[25] Shen C.-H., Tung S.-Y., Huang W.-S., Lu C.-C., Lee K.-C., Hsieh Y.-Y., Chang P.-J., Liang H.-F., Chen J.-H., Lin T.-H. (2014). Exploring the Effects of Tert-Butylhydroperoxide Induced Liver Injury Using Proteomic Approach. Toxicology.

[26] Liu Z., Du Q., Zhao J., Yang R., Yan Z., Liu C., Zhao Y. (2025). A Comparative Study of the Toxic Effects of t-BHP on AC16 and H9c2 Cardiomyocytes. J. Appl. Toxicol..

[27] Skrabalova J., Karlovska I., Hejnova L., Novotny J. (2018). Protective Effect of Morphine Against the Oxidant-Induced Injury in H9c2 Cells. Cardiovasc. Toxicol..

[28] Zhu J., Li G., Zhou J., Xu Z., Xu J. (2022). Cytoprotective Effects and Antioxidant Activities of Acteoside and Various Extracts of Clerodendrum Cyrtophyllum Turcz Leaves against T-BHP Induced Oxidative Damage. Sci. Rep..

[29] Khan A.A., Betel D., Miller M.L., Sander C., Leslie C.S., Marks D.S. (2009). Transfection of Small RNAs Globally Perturbs Gene Regulation by Endogenous microRNAs. Nat. Biotechnol..

[30] Miao S., Wan W., Hu J., Chang E., Zhou Z., Zhou Y., Sun L. (2022). Dietary Arachidonic Acid Affects the Innate Immunity, Antioxidant Capacities, Intestinal Health and Microbiota in Chinese Mitten Crab (*Eriocheir sinensis*). Aquaculture.

[31] Bradford M.M. (1976). A Rapid and Sensitive Method for the Quantitation of Microgram Quantities of Protein Utilizing the Principle of Protein-Dye Binding. Anal. Biochem..

[32] Jayashree G.V., Kumar K.H., Krupashree K., Rachitha P., Khanum F. (2015). LC–ESI–MS/MS Analysis of Asparagus Racemosus Willd. Roots and Its Protective Effects against t-BHP Induced Oxidative Stress in Rats. Ind. Crops Prod..

[33] Ochi T., Miyaura S. (1989). Cytotoxicity of an Organic Hydroperoxide and Cellular Antioxidant Defense System against Hydroperoxides in Cultured Mammalian Cells. Toxicology.

[34] Oh J.M., Jung Y.S., Jeon B.S., Yoon B.I., Lee K.S., Kim B.H., Oh S.J., Kim S.K. (2012). Evaluation of Hepatotoxicity and Oxidative Stress in Rats Treated with Tert-Butyl Hydroperoxide. Food Chem. Toxicol..

[35] Li L., Zhang Z., Huang Y. (2020). Integrative Transcriptome Analysis and Discovery of Signaling Pathways Involved in the Protective Effects of Curcumin against Oxidative Stress in Tilapia Hepatocytes. Aquat. Toxicol..

[36] Hassanein E.H.M., Sayed A.M., Hussein O.E., Mahmoud A.M. (2020). Coumarins as Modulators of the Keap1/Nrf2/ARE Signaling Pathway. Oxid. Med. Cell Longev..

[37] Loboda A., Damulewicz M., Pyza E., Jozkowicz A., Dulak J. (2016). Role of Nrf2/HO-1 System in Development, Oxidative Stress Response and Diseases: An Evolutionarily Conserved Mechanism. Cell. Mol. Life Sci..

[38] Soetikno V., Sari F.R., Lakshmanan A.P., Arumugam S., Harima M., Suzuki K., Kawachi H., Watanabe K. (2013). Curcumin Alleviates Oxidative Stress, Inflammation, and Renal Fibrosis in Remnant Kidney through the Nrf2-Keap1 Pathway. Mol. Nutr. Food Res..

[39] Liu H., Yang Y., Yu Y.-Y., Feng J.-J., Bao X.-X., Zhao J., Yu H. (2022). Astragaloside IV Improved Antioxidative Stress Capacity and Related Gene Expression of the Keap1-Nrf2 Pathway in Grass Carp (*Ctenopharyngodon idella*) Hepatocytes under Heat Stress. J. Fish Biol..

[40] Lu Y.-P., Zheng P.-H., Zhang Z.-L., Li J.-T., Li J.-J., Li T., Wang X., Xu J.-R., Wang D.-M., Xian J.-A. (2023). Effects of Dietary *Radix bupleuri* Root Extract on the Growth, Muscle Composition, Histology, Immune Responses and Microcystin-LR Stress Resistance of Juvenile Red Claw Crayfish (*Cherax quadricarinatus*). Aquac. Rep..

[41] Liu F., Shao G.-Y., Tian Q.-Q., Cheng B.-X., Shen C., Wang A.-M., Zhang J.-H., Tian H.-Y., Yang W.-P., Yu Y.-B. (2021). Enhanced Growth Performance, Immune Responses, Immune-Related Gene Expression and Disease Resistance of Red Swamp Crayfish (*Procambarus clarkii*) Fed Dietary Glycyrrhizic Acid. Aquaculture.

[42] Cai W., Zhang B., Duan D., Wu J., Fang J. (2012). Curcumin Targeting the Thioredoxin System Elevates Oxidative Stress in HeLa Cells. Toxicol. Appl. Pharmacol..

[43] Nie P., Meng F., Zhang J., Wei X., Shen C. (2019). Astragaloside IV Exerts a Myocardial Protective Effect against Cardiac Hypertrophy in Rats, Partially via Activating the Nrf2/HO-1 Signaling Pathway. Oxidative Med. Cell. Longev..

[44] Wang F., Chen S., Deng L., Chen L., Huang Y., Tian M., Li C., Zhou X. (2019). Protective Effects of Astragaloside IV against LPS-Induced Endometritis in Mice through Inhibiting Activation of the NF-κB, P38 and JNK Signaling Pathways. Molecules.

[45] Huang Y., Wang W., Xu Z., Pan J., Zhao Z., Ren Q. (2018). *Eriocheir sinensis* microRNA-7 Targets Crab *Myd88* to Enhance White Spot Syndrome Virus Replication. Fish Shellfish Immunol..

[46] Liu J., Zhang P., Wang B., Lu Y., Li L., Li Y., Liu S. (2022). Evaluation of the Effects of Astragalus Polysaccharides as Immunostimulants on the Immune Response of Crucian Carp and against SVCV in Vitro and in Vivo. Comp. Biochem. Physiol. Part C Toxicol. Pharmacol..

[47] Xu J., He J., Zhou Y.L., Weng Z., Li M., Wang Z.X., He Y. (2024). Von Willebrand Factor Promotes Radiation-Induced Intestinal Injury (RIII) Development and Its Cleavage Enzyme rhADAMTS13 Protects against RIII by Reducing Inflammation and Oxidative Stress. Free Radic. Biol. Med..

[48] Usui M., Egashira K., Kitamoto S., Koyanagi M., Katoh M., Kataoka C., Shimokawa H., Takeshita A. (1999). Pathogenic Role of Oxidative Stress in Vascular Angiotensin-Converting Enzyme Activation in Long-Term Blockade of Nitric Oxide Synthesis in Rats. Hypertension.

[49] Beck R., Dejeans N., Glorieux C., Creton M., Delaive E., Dieu M., Raes M., Levêque P., Gallez B., Depuydt M. (2012). Hsp90 Is Cleaved by Reactive Oxygen Species at a Highly Conserved N-Terminal Amino Acid Motif. PLoS ONE.

[50] Lévy E., El Banna N., Baïlle D., Heneman-Masurel A., Truchet S., Rezaei H., Huang M.-E., Béringue V., Martin D., Vernis L. (2019). Causative Links between Protein Aggregation and Oxidative Stress: A Review. Int. J. Mol. Sci..

[51] Zhu J.-W., Chen T., Guan J., Liu W.-B., Liu J. (2012). Neuroprotective Effects of Allicin on Spinal Cord Ischemia–Reperfusion Injury via Improvement of Mitochondrial Function in Rabbits. Neurochem. Int..

[52] Liu S.-G., Ren P.-Y., Wang G.-Y., Yao S.-X., He X.-J. (2015). Allicin Protects Spinal Cord Neurons from Glutamate-Induced Oxidative Stress through Regulating the Heat Shock Protein 70/Inducible Nitric Oxide Synthase Pathway. Food Funct..

[53] Darias M.J., Mazurais D., Koumoundouros G., Cahu C.L., Zambonino-Infante J.L. (2011). Overview of Vitamin D and C Requirements in Fish and Their Influence on the Skeletal System. Aquaculture.

[54] Lee M.-H., Shiau S.-Y. (2003). Increase of Dietary Vitamin C Improves Haemocyte Respiratory Burst Response and Growth of Juvenile Grass Shrimp, Penaeus Monodon, Fed with High Dietary Copper. Fish Shellfish Immunol..

[55] Gómez-Sierra T., Medina-Campos O.N., Solano J.D., Ibarra-Rubio M.E., Pedraza-Chaverri J. (2020). Isoliquiritigenin Pretreatment Induces Endoplasmic Reticulum Stress-Mediated Hormesis and Attenuates Cisplatin-Induced Oxidative Stress and Damage in LLC-PK1 Cells. Molecules.

[56] Huang W., Yao C., Liu Y., Xu N., Yin Z., Xu W., Miao Y., Mai K., Ai Q. (2020). Dietary Allicin Improved the Survival and Growth of Large Yellow Croaker (*Larimichthys crocea*) Larvae via Promoting Intestinal Development, Alleviating Inflammation and Enhancing Appetite. Front. Physiol..

